# *Naegleria fowleri:* Portrait of a Cerebral Killer

**DOI:** 10.3390/diagnostics15010089

**Published:** 2025-01-03

**Authors:** Nguyen The Nguyen Phung, Huong Thien Pham, Thuc Thanh Tran, Vu Hoang Dinh, Nhut Minh Tran, Nuong Ai Nguyen Tran, Minh Quang Ngoc Ngo, Huong Thanh Thi Nguyen, Duy Khanh Tran, Thao Kieu Thi Le, Camelia Quek, Van Hung Pham, Son Truong Pham

**Affiliations:** 1 Department of Pediatrics, University of Medicine and Pharmacy at Ho Chi Minh City, Ho Chi Minh City 700000, Vietnam; nguyenphung@ump.edu.vn (N.T.N.P.); tranthanhthuc@ump.edu.vn (T.T.T.); 2 Children’s Hospital 1, Ho Chi Minh City 700000, Vietnam; linhphuongquan@gmail.com (V.H.D.); minhnhutcttpcl@gmail.com (N.M.T.); nuongtran0206@gmail.com (N.A.N.T.); minhnnq@nhidong.org.vn (M.Q.N.N.); drnguyenhuong@gmail.com (H.T.T.N.); 3 Vietnam Research and Development Institute of Clinical Microbiology, Ho Chi Minh City 700000, Vietnam; phamthienhuong@gmail.com (H.T.P.); khanhduysh@gmail.com (D.K.T.); banglangdl1204@gmail.com (T.K.T.L.); 4 Sydney Medical School–Westmead, Sydney, NSW 2006, Australia; camelia.quek@sydney.edu.au; 5 New South Wales Health, Sydney, NSW 2065, Australia; 6 Royal Australian College of General Practitioners, Sydney, NSW 2000, Australia; 7 Australasian College for Emergency Medicine, Melbourne, VIC 3003, Australia

**Keywords:** primary amebic meningoencephalitis, multiplex real-time PCR, pediatric infectious diseases

## Abstract

**Background**: Primary amebic meningoencephalitis (PAM) caused by *Naegleria fowleri* is a rare and devastating infection of the central nervous system, often diagnosed late, due to its rapid progression and nonspecific symptoms. **Case Presentation:** We report one of the youngest documented pediatric Vietnamese cases of PAM in a 10-month-old girl from the Mekong Delta, Vietnam. The diagnosis was confirmed through multiplex real-time PCR (MPL-rPCR), microscopy, and sequencing. Clinical data were gathered retrospectively from medical records, and additional details were provided by the patient’s family. Treatment regimens, disease progression, and diagnostic challenges were reviewed and compared to existing literature. With intensive treatment, the child survived for 14 days, representing one of the longest reported pediatric PAM survival durations. No direct exposure to untreated freshwater or other typical risk factors for *Naegleria fowleri* infection was identified, underscoring the unique epidemiological nature of this case. MPL-rPCR enabled timely detection of the pathogen and demonstrated its utility in resource-limited settings. **Conclusions**: This case highlights the critical need for rapid, accessible diagnostic tools such as MPL-rPCR, particularly in resource-constrained environments where traditional diagnostics may not be feasible. It also emphasizes the importance of international collaboration and investment in cost-effective diagnostics and novel therapeutic strategies. The geographical expansion of PAM due to climate change further underscores the urgency of these measures to improve health outcomes in vulnerable populations.

## 1. Background

Primary amebic meningoencephalitis (PAM) was first identified in the 1960s [1]. It is a rare but fatal infection of the central nervous system that requires timely and accurate diagnosis for successful treatment. The main genera of ameba that can cause disease in humans include *Naegleria*, *Acanthamoeba*, *Balamuthia*, and, in one documented case, *Sappinia*. Among these, *Naegleria fowleri* is the most common cause of PAM [2]. *Naegleria fowleri* is a single-celled organism belonging to the phylum *Percolozoa*, commonly referred to as the “brain-eating ameba”. It is the only species of *Naegleria* known to cause disease in humans. Typically found in warm freshwater environments such as ponds, lakes, rivers and hot springs, this ameba thrives in temperatures above 30 °C and can tolerate temperatures up to 45 °C in free-living environments [3,4]. Infection often occurs when contaminated water enters the nasal passages, allowing the ameba to invade the brain through the nasal mucosa, resulted in PAM. PAM progresses rapidly and, without prompt treatment, the condition is almost always fatal [5].

Over 400 cases of PAM have been reported worldwide, predominantly in the United States of America (U.S.A.), Pakistan, and Australia [6,7,8]. The annual incidence in the U.S.A. is typically 0–8 cases per year, making the disease extremely rare but almost universally fatal, with a case fatality rate exceeding 97% [6,7,8]. In Vietnam, the first case of PAM was reported in 2012 [9]. *N. fowleri*, was named in honor of Malcolm Fowler, who documented the first PAM case in Australia [10].

Following entry into the body via the nose, *N. fowleri* can cause illness rapidly by invading the brain, typically within 2 to 8 days, and sometimes even sooner. Early symptoms are often nonspecific, such as fever and headache, and mimic those of more common conditions like viral or bacterial meningitis, leading to delayed or missed diagnoses. As the infection progresses and affects more brain areas, neurological symptoms emerge [11,12,13] The nonspecific nature of PAM symptoms, which resemble other forms of meningoencephalitis, hampers timely diagnosis and treatment, often leading to poor outcomes and high mortality rates.

Polymerase chain reaction (PCR) is widely regarded as the gold standard diagnostic tool for detecting microbial pathogens [14,15]. However, traditional PCR methods may focus on single pathogens, which can be time-consuming in cases where multiple potential causative agents are being considered. To address this limitation, multiplex PCR (MPL-rPCR) has emerged as a powerful extension of PCR technology. MPL-rPCR enables the simultaneous detection of multiple pathogens, including *N. fowleri*, in a single assay. This makes it especially valuable in resource-limited settings, where rapid and comprehensive diagnostic tools can significantly reduce the time to diagnosis and improve survival outcomes.

In resource-limited healthcare systems, the lack of awareness and access to advanced diagnostic techniques means that PAM is often identified only after significant disease progression, reducing the chances of survival. Historically, diagnostic methods for PAM have included clinical examination, cerebrospinal fluid (CSF) microscopy, and culture. However, due to the rapid progression and nonspecific initial symptoms, early diagnosis has remained challenging. The application of MPL-rPCR as a rapid, sensitive and specific diagnostic tool is critical in these contexts, particularly for the early detection of rare infections such as PAM, even at low pathogen loads, enabling rapid confirmation of infection. Guidelines from the Centers for Disease Control and Prevention (CDC) recommend PCR as the definitive diagnostic method in suspected PAM cases, supplementing traditional microscopy and culture techniques. Recent reviews emphasize that PCR-based assays have significantly improved diagnostic accuracy in both clinical and research settings, particularly in resource-limited environments.

In this case report, we present the first pediatric case of PAM caused by *N. fowleri* in a child treated at Children’s Hospital 1, one of three largest pediatric healthcare facilities in southern Vietnam. The implementation of MPL-rPCR as a first-line diagnostic tool, as demonstrated in this case, could be life-saving, not only in detecting PAM but in guiding treatment for other forms of meningoencephalitis as well.

## 2. Case Presentation

**Figure 1:**
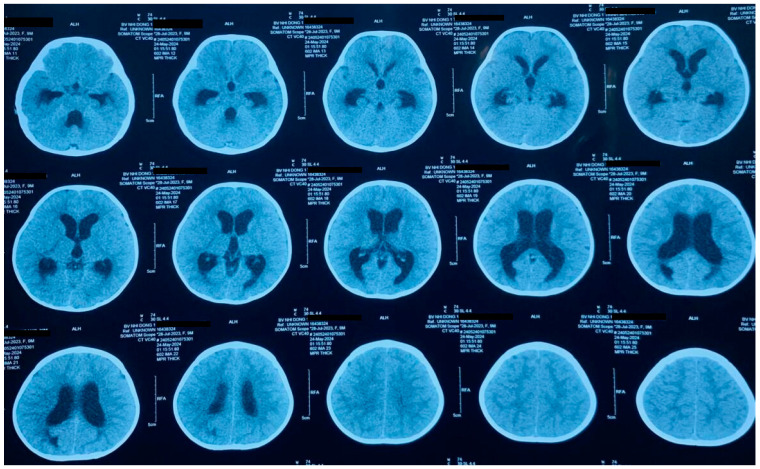
Cranial CT scan images of the 10-month-old patient demonstrating acute hydrocephalus. The scans show significant enlargement of the lateral ventricles due to increased intracranial pressure caused by severe cerebral edema. These findings are consistent with advanced primary amebic meningoencephalitis (PAM), highlighting the rapid progression of the disease and its impact on the central nervous system.

A previously healthy 10-month-old child, living in Mekong delta, Vietnam, presented to the Children’s Hospital 1 in Ho Chi Minh City, Vietnam, with a 3-day history of worsening symptoms, including high grade fevers, frequent vomiting, and lethargy, without apparent signs of meningism, trauma or contact with sick people. Her antenatal and postnatal history was unremarkable. Upon arrival at the emergency department, she was given ceftriaxone (100 mg/kg) as empirical treatment for sepsis of unknown origin and promptly admitted to the General Medicine department to continue active treatment and investigations. Within 8 h post-admission on day 4, the patient experienced multiple generalized seizures accompanied by reduced level of consciousness (U on AVPU), necessitating intubation. Following intubation, the child’s condition deteriorated, leading to a deep coma, with no response to stimuli. The patient underwent intensive management, including mechanical ventilation, intravenous administration of meropenem and vancomycin for broad-spectrum antimicrobial therapy, intracranial pressure control with intravenous mannitol and hypertonic saline, and emergency external ventricular drainage to address severe cerebral edema. Brain ultrasound and computed tomography (CT) scans revealed acute hydrocephalus (Figure 1).

Laboratory tests showed an elevated C-reactive protein (CRP) level of 151 mg/L. Urinalysis and urine microscopy culture-sensitivity were negative. The progress of the patient’s laboratory parameters is presented in Table 1, showing worsening systemic and central nervous system inflammation and disease progress, with raised CRP at the expense of white blood counts (WBC). Chest X-Ray and abdominal ultrasound findings were unremarkable, while the fontanel ultrasound showed hydrocephalus. Cerebrospinal fluid (CSF) analysis showed a turbid yellow appearance with 4.032 × 10^3^ white cells/mL (88% polymorphonuclear leukocytes), CSF lactate of 11.8 mmol/L, and CSF protein of 6.9 g/L. Plasma glucose was always within normal range. Subsequent blood and CSF bacterial microscopy and culture were negative. All other diagnostic tests, for common encephalitis pathogens in Vietnam such as herpes simplex virus, Japanese encephalitis virus and *Mycobacterium tuberculosis*, were also negative. Auto-immune indicators, such as antibody, and complement levels were all within normal limits. The anti-HIV result was also negative.

The differential diagnoses for a child presenting with a 3-day history of worsening fever, vomiting and lethargy are broad. Specifically, fever is suggestive of infection, particularly in infants, thus, viral gastroenteritis, urinary tract infection and sepsis of unknown origin can all present with nonspecific symptoms, such as vomiting and lethargy, without localized signs. Notably, early symptoms of meningitis can also be nonspecific, before more severe neurological signs appear. Although less likely, intussusception should also be considered as a possible surgical cause for this child presentation, as it can present with vomiting and lethargy, and sometimes fever, especially in this child’s age group.

The CSF was turbid and yellow, indicated abnormal findings with high protein content and/or the elevated white blood cell (WBC) count of 4032 cells/mm^3^, with 88% polymorphonuclear leukocytes (PMNs). This marked neutrophil-predominant pleocytosis is characteristic of bacterial meningitis, but can also occur in other severe infections or inflammatory conditions like amebic meningoencephalitis. The protein level was also significantly elevated at 6.9 g/L, well above the normal range (typically 0.15–0.45 g/L), suggesting bacterial or severe viral infections, or in conditions like Guillain-Barré syndrome. While the specific CSF/serum glucose ratio was not provided, a significantly low CSF glucose concentration (relative to blood glucose) would be highly suggestive of infectious meningitis. Additionally, the lactate level was high at 11.8 mmol/L, often a result of anaerobic metabolism due to infection, particularly bacterial, though it can also be elevated in cases of severe CNS infection like amebic meningoencephalitis. Consequently, the significantly high white cell counts and elevated protein and lactate levels are consistent with the severe inflammatory response seen in PAM. Due to the severity and vague presentation, it is also essential to consider and test for all potential causative agents, including rare pathogens, to ensure a comprehensive diagnosis.

Given the rapid clinical deterioration and CSF findings in the absence of confirmatory microscopic and culture results, MPL-rPCR was performed on day 4 of disease onset. The MENINGIcheck diagnostic panel, developed by the Vietnam Research and Development Institute of Clinical Microbiology, was employed to detect a wide spectrum of pathogens associated with meningoencephalitis, including bacteria, viruses, fungi, and parasites (Supplementary Table S1).

For this procedure, DNA/RNA was extracted from 200 µL of CSF using the ^NK^DNARNAprep-MAGBEAD extraction kit (Nam Khoa Company, Ho Chi Minh City, Vietnam). The extracted nucleic acids were then subjected to real-time PCR using the MENINGIcheck panel, which targets specific genetic markers of pathogens. Amplification was carried out on a CFX-96 PCR machine (Bio-Rad, Hercules, CA, USA) under the following cycling conditions: reverse transcription at 45 °C for 10 min, enzyme inactivation at 95 °C for 10 min, followed by 40 amplification cycles consisting of denaturation at 95 °C for 15 s and annealing/extension at 60 °C for 1 min. Fluorescence signals were recorded during the annealing/extension phase.

MENINGIcheck is a novel diagnostic panel designed for pathogen identification by employing primers and probes specific to the genetic markers of relevant pathogens. Where existing primers and probes were available from published sources, they were incorporated into the panel [16,17,18,19,20]. For pathogens lacking prior designs, specific gene sequences were retrieved from GenBank, and custom primers and probes were developed. This systematic approach ensures comprehensive detection of pathogens relevant to meningitis.

**Figure 2:**
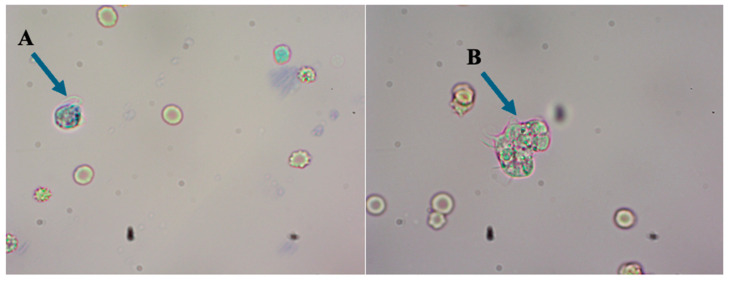
The “brain-eating” ameba in CSF were detected by direct microscopic examination under the wet preparation that showed the flagellated form (**A**) and the trophozoites form (**B**). Original magnification ×40.

Notably, this test detected a high copy number of *N. fowleri* target DNA with Ct = 21.92 (Supplementary Figure S1) and was negative for other pathogens. This result was subsequently confirmed by direct microscopic examination, which revealed the presence of the “brain-eating” ameba in the CSF sample (Figure 2). Sanger sequencing of the specific DNA sequence in the 18S ribosomal RNA gene of *N. fowleri* was also used to confirm the detection by MPL-rPCR (Supplementary Figures S2 and S3). This result guided us to give immediate agent-specific treatments as per the Centers for Disease Control and Prevention (CDC, U.S.A.) protocol, including fluconazole (10 mg/kg), amphotericin B (1.5 mg/kg), rifampicin (10 mg/kg), azithromycin (10 mg/kg), and dexamethasone (0.6 mg/kg), in combination with broad-spectrum antibiotics including meropenem (120 mg/kg) and linezolid (30 mg/kg). Despite all the active treatment, the patient’s condition continued to be critical and deteriorate and she passed away after 11 days of hospitalization.

## 3. Discussion

We report one of the youngest, rapidly deteriorating, fatal cases of pediatric PAM caused by *Naegleria fowleri* infection in a previously healthy 10-month-old child with no known risk factors or prior exposures, from the Mekong Delta, Vietnam, who required intensive care. MPL-rPCR and direct microscopy were used to confirm the diagnosis. Despite the intensive treatment, the child passed away 14 days after disease onset. We discuss the diagnostic challenges and current treatments for this fatal infection.

Early diagnosis of PAM is critical, yet extremely challenging, due to the nonspecific symptoms of PAM in its initial stages, which resemble bacterial or viral meningitis [21], together with limited diagnostic tools, including CSF analysis, PCR and direct microscopy [19,21,22]. Specifically, CSF typically shows a neutrophilic pleocytosis, with elevated protein and low glucose levels, although these findings are nonspecific, and can lead to diagnostic uncertainty. Moreover, PCR for *Naegleria fowleri* DNA is a sensitive and specific test that can confirm the diagnosis rapidly, yet it is not routinely requested unless the disease is suspected. The lack of routinely available PCR tests, except in specialized pathology laboratories, further compounds the problem in resource-constrained environments. Wet mount microscopy of CSF can sometimes reveal motile trophozoites, but this is less commonly used, due to the need for specialized expertise. In this case, only Gram stain, cultures and CSF cytology, together with biochemical tests, were the routine initial tests for suspected meningoencephalitis, which were all negative, highlighting the need for early access to more reliable and accessible diagnostic tools in such settings.

The use of MPL-rPCR in this case was invaluable, demonstrating its capability to detect a wide range of pathogens simultaneously, even in cases where the cause of meningoencephalitis is uncertain (Supplementary Table S1). This technique is designed to be accessible, utilizing publicly available sequence data from GenBank for in-house development by local laboratories, which is particularly important in low-resource settings. Although the initial costs may be higher, compared to traditional diagnostics, economies of scale achieved through global standardization can drive costs down. This assay’s ability to provide rapid, simultaneous detection of multiple pathogens is especially valuable in resource-limited settings, where its implementation can significantly improve diagnostic outcomes and reduce healthcare burdens. Future advancements, such as whole-genome sequencing, are proposed as complementary technologies. Furthermore, the potential for international collaborations to facilitate the widespread adoption of MPL-rPCR is emphasized, including partnerships to improve affordability and accessibility. The MPL-rPCR addresses a critical limitation in current diagnostic practices by broadening the scope beyond common pathogens that are often presumed to be present, based on clinical presentation and patient risk factors. This comprehensive coverage helps mitigate the risks of misdiagnosis or missed diagnoses, especially for uncommon or unexpected pathogens, as demonstrated in this case. For regions with diverse pathogen profiles or limited access to specialized diagnostic tools, MPL-rPCR offers a transformative solution.

Moreover, this assay’s rapid and reliable performance makes it well suited for emergency settings, enabling the early detection of pathogens and timely interventions that could significantly improve survival rates in cases of PAM and other infectious diseases. By equipping local laboratories with the means to develop and implement such tools, MPL-rPCR holds the potential to revolutionize diagnostic approaches for meningitis and other complex infectious diseases on a global scale.

Once diagnosed, the treatment for PAM remains a challenge, due to the rapid progression of the disease, together with delayed initial diagnosis, and the lack of strong evidence-based guidelines for these cases [8]. The most commonly used regimen includes a combination of intravenous amphotericin B, rifampin, fluconazole, miltefosine, and azithromycin [23,24]. Amphotericin B is the most well-established treatment for PAM, due to its direct amebicidal effect. It binds to ergosterol in the cell membranes of *N. fowleri*, causing increased membrane permeability and cell death [25]. In fact, several case reports and small case series have shown that aggressive treatment with amphotericin B, particularly when initiated early, can improve survival, although the overall mortality remains high [25,26,27]. Rifampicin is a broad-spectrum antibiotic with activity against many bacterial species and some evidence of efficacy against *N. fowleri* in vitro [28]. Its use in PAM is based on in vitro studies and case reports, with no strong clinical trial data to support its efficacy [29,30]. Likewise, fluconazole is an antifungal agent that inhibits fungal sterol synthesis. While not as potent as amphotericin B, it has been used as an adjunct therapy, due to its ability to cross the blood–brain barrier [3,29,31]. Its use is based on a few case reports suggesting some benefit, particularly when used in combination with other therapies [3,28,29]. In addition to the aforementioned drugs, azithromycin, a macrolide antibiotic, has shown some in vitro activity against *N. fowleri*. It is thought to work by inhibiting protein synthesis in the ameba [3,32]. Like rifampicin and fluconazole, azithromycin is used based on limited evidence from case studies, often as part of a combination therapy [3,32,33].

Consequently, the rapid onset and progression of PAM leave little time for intervention. This, combined with the initial nonspecific symptoms, often leads to delays in diagnosis and treatment [3,4]. This highlights the importance of routine application of broad-spectrum diagnostic tools, like the MLP-rPCR for this case, which provide a significant advantage, due to their speed, robustness, and ability to detect a wide range of pathogens at the same time. Such tools are invaluable and should be implemented as early as the presentation to the emergency department for undifferentiated septic cases for early diagnosis, where time-sensitive, accurate detection and intervention is critical.

Despite intensive treatment, the mortality rate for PAM remains extremely high, exceeding 95% in most cases [4,34]. The few documented survivors typically received early, aggressive therapy and may have had less severe initial presentations [28,35]. Nevertheless, the rarity of PAM makes large-scale clinical trials difficult, so most treatment recommendations are based on case reports and expert consensus rather than high-quality randomized controlled trials [27,36]. Beyond the standard treatments, research is ongoing into new therapeutic approaches, including therapeutic hypothermia and novel pharmacological agents. However, these remain experimental, and further research is needed to establish their efficacy [27,37].

Looking to the future, there is increasing interest in the development of vaccines or other preventive measures for *Naegleria fowleri* infections, though these are still in the early stages of research [38,39]. Conversely, efforts to improve the accessibility of diagnostic tools like MPL-rPCR should be prioritized. Collaborative international efforts between research institutions, governments, and global health organizations are essential to make these diagnostic technologies available in resource-limited settings. The case described in this article demonstrates how international collaboration and the integration of advanced diagnostic tools can enhance early diagnosis, improve treatment outcomes, and ultimately reduce the global burden of rare but deadly infections like PAM.

This case of primary amebic meningoencephalitis (PAM) is unique, in that no direct risk factors or typical exposure pathways for *Naegleria fowleri* were identified. Despite thorough inquiries with the deceased infant’s parents and next of kin, there were no reported exposures to untreated freshwater sources, recreational water activities, or other commonly recognized risk factors. However, considering the ecology of *Naegleria fowleri*, it is plausible that the infant was exposed indirectly through household water use. *Naegleria fowleri* thrives in warm freshwater, including inadequately treated or untreated water supplies. Possible exposure could have occurred during bathing, nasal rinsing, or other contact with contaminated household water. While such pathways are less frequently reported compared to recreational water activities, they represent a potential risk that warrants further investigation. By addressing these considerations, this case report aims to raise awareness among healthcare professionals and the public about the challenges of diagnosing and mitigating such infections.

The global health implications of this case are significant. Climate change and rising water temperatures are likely to increase the geographical spread of *Naegleria fowleri*, particularly in tropical and subtropical regions. As these environmental changes take place, it is essential to develop surveillance systems and improve awareness about PAM in at-risk areas. Strengthening diagnostic capabilities, particularly with multiplex PCR, and ensuring that treatment options are accessible in resource-poor settings will be crucial to mitigating the impact of PAM on vulnerable populations. By focusing on these strategies, healthcare systems can be better prepared to address this rare but devastating infection.

## 4. Conclusions

This case, one of the youngest reported for *Naegleria fowleri* infection, highlights the critical need for rapid, accessible diagnostic tools like MPL-rPCR, especially in resource-limited settings. PAM’s rapid progression and poor survival rates demand early diagnosis, as traditional methods often fail in time-sensitive scenarios. Despite intensive treatment with amphotericin B, often in combination with other agents like miltefosine, fluconazole, rifampicin, and azithromycin, alongside aggressive supportive care, the survival rate remains low, underscoring the need for urgent research into novel therapies. It is concluded that routine implementation of MPL-rPCR and global collaboration are essential to combat this deadly disease more effectively.

## Figures and Tables

**Table 1:** The patient’s progress in investigations with timeline since symptom onset.

Parameters	Admission Day 1 Disease Progress Day 3	Admission Day 3 Disease Progress Day 5
WBC (10^3^/μL)	13.4	5.69
NEU (10^3^/μL)	10.1	4.72
LYM (10^3^/μL)	2.0	0.79
Hb (g/dL)	9.9	10.8
PLT (10^3^/μL)	264	151
CRP (mg/L)	151	179.2
AST (U/L)	38.2	91.5
ALT (U/L)	11.9	32.3
Creatinine (μmol/L)	32	25.8
CSF cells (cells/mm^3^)	4032 (88% polymorphonuclear leukocytes)	962 (85% polymorphonuclear leukocytes)
CSF/serum glucose (%)	26	66
CSF lactate (mmol/L)	11.8	10.3
CSF protein (g/L)	32.4	34.3

Abbreviation: WBC: white blood cells; NEU: neutrophils; LYM: lymphocytes; Hb: hemoglobin; PLT: platelets; CRP: C-reactive protein; AST: aspartate aminotransferase; ALT: alanine aminotransferase; CSF: cerebrospinal fluid.

## Data Availability

The datasets generated and/or analyzed during the current study are available from the corresponding authors on reasonable request.

## Supplementary Materials

The following supporting information can be downloaded at: https://www.mdpi.com/article/10.3390/diagnostics15010089/s1, **Figure S1.** The MPL-rPCR result showed the high copy number of *Naegleria fowleri* target DNA with Ct = 21.92 (pink color). The amplification curve (green color) is of the patient’s human beta-globin to check the quality of the sample; **Figure S2.** The Sanger sequencing result confirmed the pathogen detected in the CSF sample collected from the patient was the *Naegleria fowleri*. BLAST analysis against GenBank revealed more than 99.46% similarity with previously reported *Naegleria fowleri* sequences. The sequence has been submitted to the GenBank database with the accession number PQ740299; **Figure S3.** The phylogenetic analysis showed that the detected *Naegleria fowleri* is in the same branch as ATCC30894 and the Karachi NF001 strain. This bootstrap was performed by the maximum likelihood method; **Table S1.** List of pathogens detectable in the multiplex real-time PCR (MPL-rPCR).

## References

[1] Duma R.J., Ferrell H.W., Nelson E.C., Jones M.M. (1969). Primary amebic meningoencephalitis. N. Engl. J. Med..

[2] Qvarnstrom Y., da Silva A.J., Schuster F.L., Gelman B.B., Visvesvara G.S. (2009). Molecular confirmation of *Sappinia pedata* as a causative agent of amoebic encephalitis. J. Infect. Dis..

[3] Grace E., Asbill S., Virga K. (2015). *Naegleria fowleri*: Pathogenesis, diagnosis, and treatment options. Antimicrob. Agents Chemother..

[4] Jahangeer M., Mahmood Z., Munir N., Waraich U., Tahir I.M., Akram M., Shah S.M.A., Zulfqar A., Zainab R. (2020). *Naegleria fowleri*: Sources of infection, pathophysiology, diagnosis, and management; a review. Clin. Exp. Pharmacol. Physiol..

[5] Marciano-Cabral F., Cabral G.A. (2007). The immune response to *Naegleria fowleri* amebae and pathogenesis of infection. FEMS Immunol. Med. Microbiol..

[6] Maciver S.K., Piñero J.E., Lorenzo-Morales J. (2019). Is *Naegleria fowleri* an Emerging Parasite?. Trends Parasitol..

[7] Ghanchi N.K., Jamil B., Khan E., Ansar Z., Samreen A., Zafar A., Hasan Z. (2017). Case Series of Naegleria fowleri Primary Ameobic Meningoencephalitis from Karachi, Pakistan. Am. J. Trop. Med. Hyg..

[8] Güémez A., García E. (2021). Primary Amoebic Meningoencephalitis by *Naegleria fowleri*: Pathogenesis and Treatments. Biomolecules.

[9] Phu N.H., Mai N.T.H., Nghia H.D.T., Chau T.T.H., Loc P.P., Thai L.H., Phuong T.M., Thai C.Q., Man D.N.H., Chau N.V.V. (2013). Fatal consequences of freshwater pearl diving. Lancet.

[10] Fowler M., Carter R.F. (1965). Acute pyogenic meningitis probably due to *Acanthamoeba* sp.: A preliminary report. BMJ.

[11] Gharpure R., Bliton J., Goodman A., Ali I.K.M., Yoder J., Cope J.R. (2020). Epidemiology and Clinical Characteristics of Primary Amebic Meningoencephalitis Caused by *Naegleria fowleri*: A Global Review. Clin. Infect. Dis..

[12] Cooper A.M., Aouthmany S., Shah K., Rega P.P. (2019). Killer amoebas: Primary amoebic meningoencephalitis in a changing climate. J. Am. Acad. Physician Assist..

[13] Zamzuri M.I.A., Majid F.N.A., Mihat M., Ibrahim S.S., Ismail M., Aziz S.A., Mohamed Z., Rejali L., Yahaya H., Abdullah Z. (2023). Systematic Review of Brain-Eating Amoeba: A Decade Update. Int. J. Environ. Res. Public Health.

[14] Cope J.R., Ali I.K. (2016). Primary amebic meningoencephalitis: What have we learned in the last 5 years?. Curr. Infect. Dis. Rep..

[15] Rojo J.U., Rajendran R., Salazar J.H. (2023). Laboratory Diagnosis of Primary Amoebic Meningoencephalitis. Lab. Med..

[16] Albuquerque R.C., Moreno A.C.R., Dos Santos S.R., Ragazzi S.L.B., Martinez M.B. (2019). Multiplex-PCR for diagnosis of bacterial meningitis. Braz. J. Microbiol..

[17] Kusum S., Aman S., Pallab R., Kumar S.S., Manish M., Sudesh P., Subhash V., Meera S. (2011). Multiplex PCR for rapid diagnosis of tuberculous meningitis. J. Neurol..

[18] Uzuka R., Kawashima H., Hasegawa D., Ioi H., Amaha M., Kashiwagi Y., Takekuma K., Hoshika A., Chiba K. (2004). Rapid diagnosis of bacterial meningitis by using multiplex PCR and real time PCR. Pediatr. Int..

[19] Qvarnstrom Y., Visvesvara G.S., Sriram R., Da Silva A.J. (2006). Multiplex real-time PCR assay for simultaneous detection of *Acanthamoeba* spp., *Balamuthia mandrillaris*, and *Naegleria fowleri*. J. Clin. Microbiol..

[20] Piccirilli G., Chiereghin A., Gabrielli L., Giannella M., Squarzoni D., Turello G., Felici S., Vocale C., Zuntini R., Gibertoni D. (2018). Infectious meningitis/encephalitis: Evaluation of a rapid and fully automated multiplex PCR in the microbiological diagnostic workup. New Microbiol..

[21] Yoder J.S., Eddy B.A., Visvesvara G.S., Capewell L., Beach M.J. (2009). The epidemiology of primary amoebic meningoencephalitis in the USA, 1962–2008. Epidemiol. Infect..

[22] Kiderlen A.F., Laube U. (2004). Balamuthia mandrillaris, an opportunistic agent of granulomatous amebic encephalitis, infects the brain via the olfactory nerve pathway. Parasitol. Res..

[23] Schuster F.L., Visvesvara G.S. (2004). Opportunistic amoebae: Challenges in prophylaxis and treatment. Drug Resist. Updates.

[24] CDC (2020). Treatment of Naegleria Fowleri Infections.

[25] Martinez A.J., Visvesvara G.S. (1997). Free-living, amphizoic and opportunistic amebas. Brain Pathol..

[26] Visvesvara G.S., Moura H., Schuster F.L. (2007). Pathogenic and opportunistic free-living amoebae: *Acanthamoeba* spp., *Balamuthia mandrillaris*, *Naegleria fowleri*, and *Sappinia diploidea*. FEMS Immunol. Med. Microbiol..

[27] Cope J.R., Conrad D.A., Cohen N., Cotilla M., DaSilva A., Jackson J., Visvesvara G.S. (2015). Use of the Novel Therapeutic Agent Miltefosine for the Treatment of Primary Amebic Meningoencephalitis: Report of 1 Fatal and 1 Surviving Case. Clin. Infect. Dis..

[28] Vargas-Zepeda J., Gómez-Alcalá A.V., Vázquez-Morales J.A., Licea-Amaya L., De Jonckheere J.F., Lares-Villa F. (2005). Successful treatment of *Naegleria fowleri* meningoencephalitis by using intravenous amphotericin B, fluconazole and rifampicin. Arch. Med. Res..

[29] Jarolim K.L., McCosh J.K., Howard M.J., John D.T. (2000). A light microscopy study of the migration of Naegleria fowleri from the nasal submucosa to the central nervous system during the early stage of primary amebic meningoencephalitis in mice. J. Parasitol..

[30] Mungroo M.R., Khan N.A., Siddiqui R. (2019). *Naegleria fowleri*: Diagnosis, treatment options and pathogenesis. Expert Opin. Orphan Drugs.

[31] Tiewcharoen S., Junnu V., Chinabut P. (2002). In vitro effect of antifungal drugs on pathogenic *Naegleria* spp.. Southeast Asian J. Trop. Med. Public Health.

[32] Goswick S.M., Brenner G.M. (2003). Activities of azithromycin and amphotericin B against Naegleria fowleri in vitro and in a mouse model of primary amebic meningoencephalitis. Antimicrob. Agents Chemother..

[33] Kim J.-H., Jung S.-Y., Lee Y.-J., Song K.-J., Kwon D., Kim K., Park S., Im K.-I., Shin H.-J. (2008). Effect of therapeutic chemical agents in vitro and on experimental meningoencephalitis due to *Naegleria fowleri*. Antimicrob. Agents Chemother..

[34] Martínez-Castillo M., Cárdenas-Zúñiga R., Coronado-Velázquez D., Debnath A., Serrano-Luna J., Shibayama M. (2016). *Naegleria fowleri* after 50 years: Is it a neglected pathogen?. J. Med. Microbiol..

[35] Linam W.M., Ahmed M., Cope J.R., Chu C., Visvesvara G.S., da Silva A.J., Qvarnstrom Y., Green J. (2015). uccessful treatment of an adolescent with Naegleria fowleri primary amebic meningoencephalitis. Pediatrics.

[36] Hall A.D., Kumar J.E., Golba C.E., Luckett K.M., Bryant W.K. (2024). Primary amebic meningoencephalitis: A review of *Naegleria fowleri* and analysis of successfully treated cases. Parasitol. Res..

[37] Heggie T.W., Küpper T. (2017). Surviving *Naegleria fowleri* infections: A successful case report and novel therapeutic approach. Travel Med. Infect. Dis..

[38] Naveed M., Ali U., Aziz T., Jabeen K., Arif M.H., Alharbi M., Alasmari A.F., Albekairi T.H. (2024). Development and immunological evaluation of an mRNA-based vaccine targeting *Naegleria fowleri* for the treatment of primary amoebic meningoencephalitis. Sci. Rep..

[39] John D.T., Weik R.R., Adams A.C. (1977). Immunization of mice against *Naegleria fowleri* infection. Infect. Immun..

