# Supplementary material for: Naegleria fowleri: Portrait of a Cerebral Killer
Source: Diagnostics (Basel). 2025 Jan 3;15(1):89. doi: 10.3390/diagnostics15010089 (PMC11719733; doi:10.3390/diagnostics15010089)
Supplement: Supplementary file 1 [file diagnostics-15-00089-s001.zip › diagnostics-3320035-supplementary.pdf]

diagnostics

Supplementary Materials

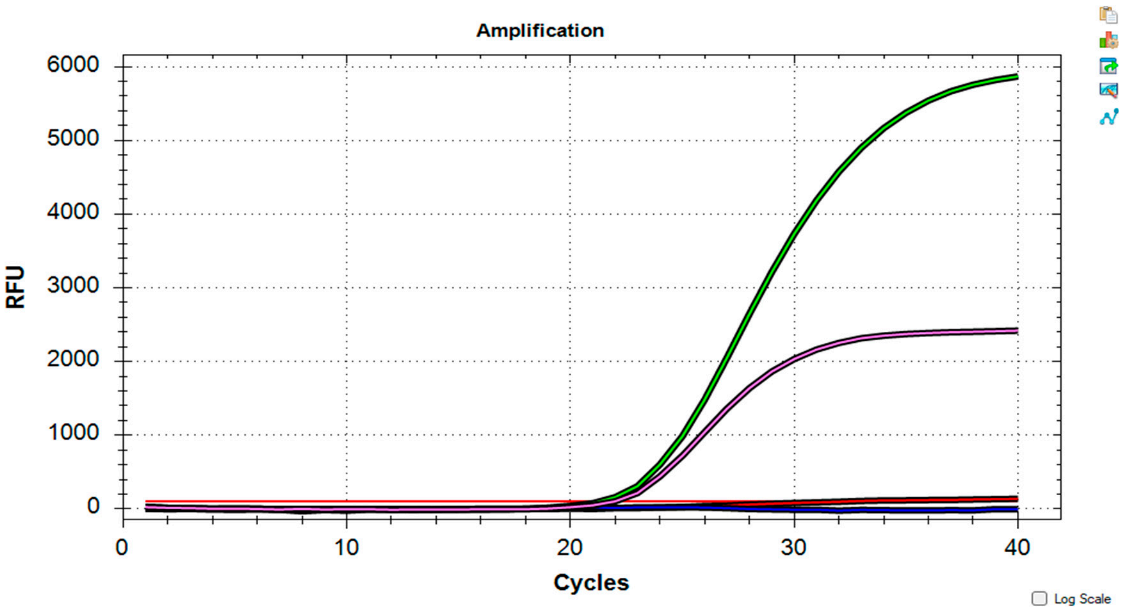

Figure S1. The MPL-rPCR result showed the high copy number of *Naegleria fowleri* target DNA with Ct = 21.92 (pink color). The amplification curve (green color) is of the patient's human beta-globin to check the quality of the sample.

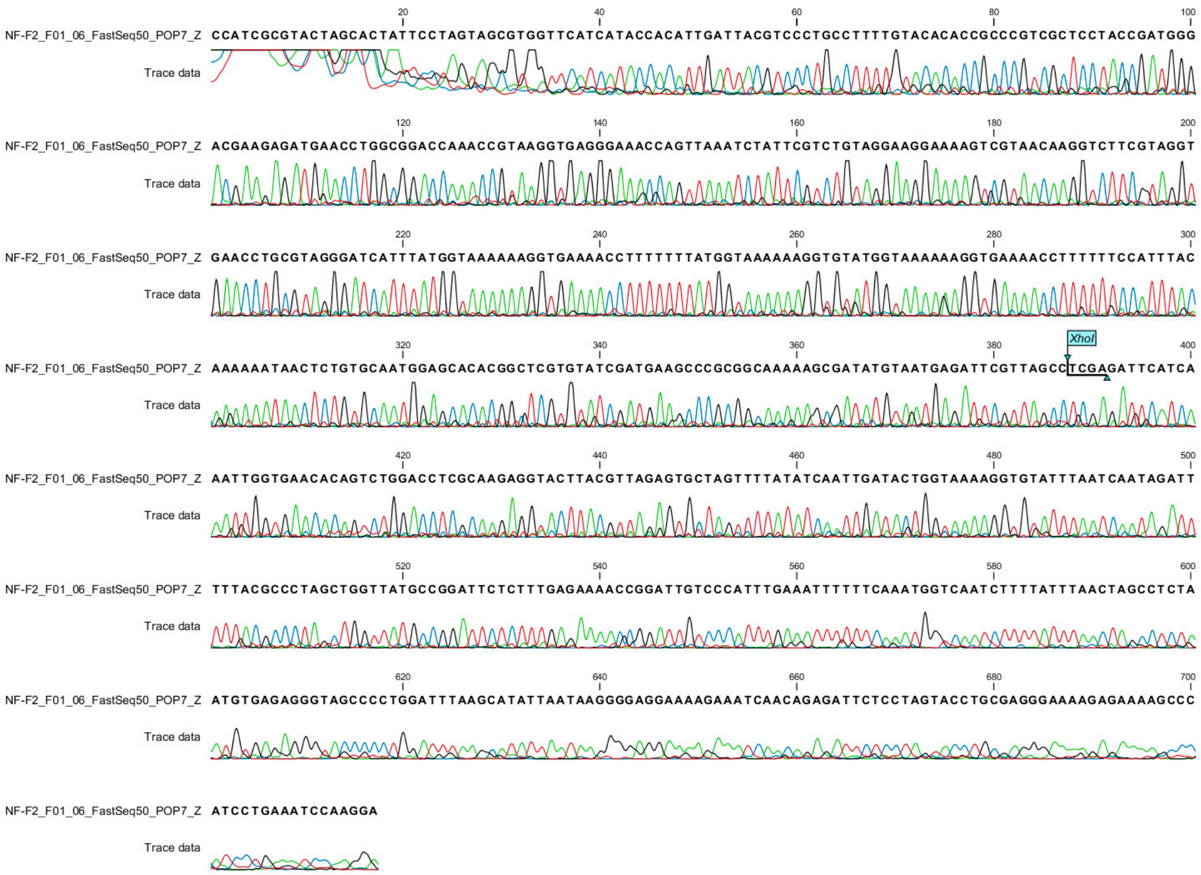

Figure S2. The Sanger sequencing result confirmed the pathogen detected in the CSF sample collected from the patient was the *Naegleria fowleri*. BLAST analysis against GenBank revealed more than 99.46% similarity with previously reported *Naegleria fowleri* sequences. The sequence has been submitted to the GenBank database with the accession number PQ740299.

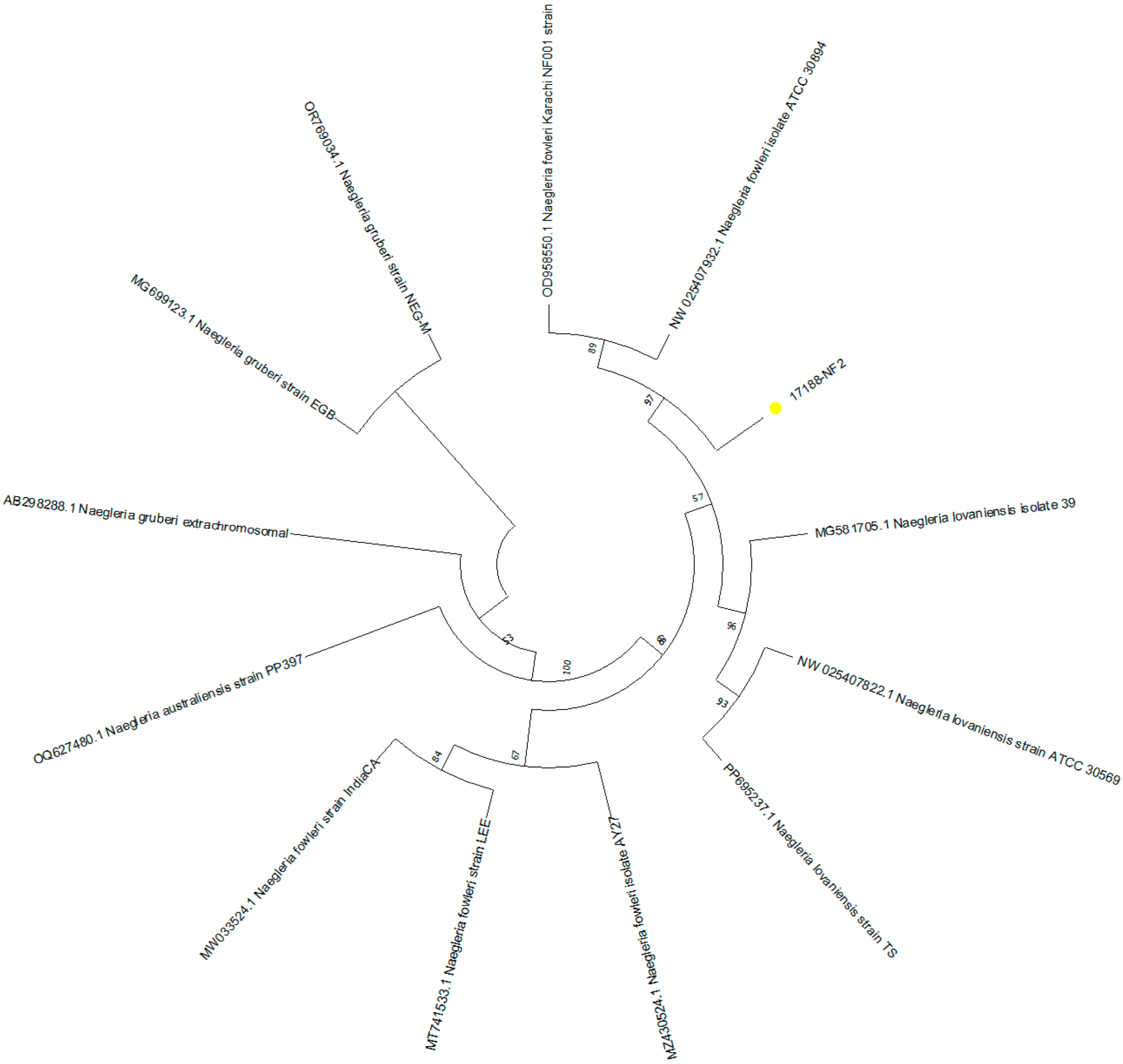

Figure S3. The phylogenetic analysis showed that the detected *Naegleria fowleri* is in the same branch as ATCC30894 and the Karachi NF001 strain. This bootstrap was performed by the maximum likelihood method.

Table S1. List of pathogens detectable in the multiplex real-time PCR (MPL-rPCR).

MPL1 (Bacteria)	MPL2 (Bacteria)	MPL3 (Bacteria)	MPL4 (Bacteria)	MPL5 (Tuberculosis)	
N. meningitidis—FAM	*Salmonella*—FAM	*S. agalactiae* (GBS)—FAM	*Leptospira interogans*—FAM	*M. tuberculosis*—FAM	
S. pneumoniae—HEX	*E. meningoseptica*—HEX	*S. pyogenes* (GAS)—HEX	*Rickettsia prowazekii*—HEX	*Nocardia asteroides*—HEX	
S. suis—TexasRED	*T. pallidum*—TexasRED	*L. monocytogenes*—TexasRED	*Brucella*—TexasRED	NTM—TexasRED	
H. influenzae type B—CY5	*E. coli* K1—CY5	*E. coli*—CY5	*Orientia tsutsugamushi*—CY5	HBG (IC)—CY5	
MPL6 (Viruses)	MPL7 (Viruses)	MPL8 (Viruses)	MPL9 (Viruses)	MPL10 (Viruses)	MPL11 (Viruses)
HSV 1—FAM	CMV—FAM	EV71—FAM	JBE—FAM	Rabie Viruses—FAM	B19—FAM
HSV 2—HEX	Varicella/Zoster Viruses—HEX	EV (EchoViruses, CoxackieViruses A,B)—HEX	Influenza Viruses A—HEX	ZikaViruses—HEX	JCV—HEX
LymphoCHORIO GP—TexasRED	EBV—TexasRED	Saffold Viruses—TexasRED	Measle Viruses—TexasRED	MUMP—TexasRED	BKV—TexasRED
LymphoCHORIO NP2—CY5	HHV6—CY5	Human ParechoViruses—CY5	Rubella Viruses—CY5	RNAseP (IC)—CY5	AdenoViruses—CY5
MPL12 (Parasites)	MPL13 (Parasites)	MPL14 (Parasites)		MPL15 (Fungi)	MPL16 (Fungi)
Acanthamoeba—FAM	*E. histolytica*—FAM	*Angiostrongylus cantonensis*—FAM		*C. neoformans*—FAM	*Coccidioides immitis/posadasii*—FAM
Naegleria fowleri—HEX	*Baylisascaris procyonis*—HEX	*Strongyloides stercoralis*—HEX		*P. jiroveci*—HEX	*Sporothrix globosa*—HEX
Balamuthia mandrillaris—TexasRED	*T. gondii*—TexasRED	*Taenia solium*—TexasRED		*Actinomyces israelii*—TexasRED	*Sporothrix schenckii/brasiliensis*—TexasRED
HBG (IC)—CY5	*Toxocara* CANIS—CY5	*Gnathostoma spinigerum*—CY5		*P. marneffei*—CY5	*Mucormycosis (Rhizopus oryzae)*—CY5

Footnote: The multiplex real-time PCR (MPL-rPCR) assay was designed and developed in-house by Vietnam Research and Development Institute of Clinical Microbiology. Supplementary Table S1 provides a comprehensive list of pathogens that can be detected. The assay consists of 16 multiplex real-time PCR mixes, each containing up to four probes, with each probe targeting a specific pathogen. This design enables simultaneous detection of multiple microorganisms, covering bacteria, viruses, parasites, and fungi. For positive controls, synthetic oligonucleotides corresponding to target gene sequences were used, rather than strains isolated from clinical samples. These oligos were designed based on reference sequences available in GenBank and serve as robust controls for each target pathogen.
